# Do RNA modifications contribute to modulation of immune responses in allergic diseases?

**DOI:** 10.3389/falgy.2023.1277244

**Published:** 2023-11-13

**Authors:** Pavel Kudrin, Ana Rebane

**Affiliations:** Institute of Biomedicine and Translational Medicine, University of Tartu, Tartu, Estonia

**Keywords:** RNA modifications, allergy, m6A, atopic dermatitis, asthma, A-to-I editing

## Abstract

RNA modifications have emerged as a fundamental mechanism of post-transcriptional gene regulation, playing vital roles in cellular physiology and the development of various diseases. While the investigation of RNA modifications has seen significant advancements, the exploration of their implication in allergic diseases has been comparatively overlooked. Allergic reactions, including hay fever, asthma, eczema and food allergies, result from hypersensitive immune responses, affecting a considerable population worldwide. Despite the high prevalence, the molecular mechanisms underlying these responses remain partially understood. The potential role of RNA modifications in modulating the hypersensitive immune responses has yet to be fully elucidated. This mini-review seeks to shed light on potential connections between RNA modifications and allergy, highlighting recent findings and potential future research directions. By expanding our understanding of the complex interplay between RNA modifications and allergic responses, we hope to unlock new avenues for allergy diagnosis, prognosis, and therapeutic intervention.

## Introduction

Canonical residues within RNA molecule can be modified in various ways including the addition of chemical group to either a base or a sugar, or both, isomerization, deamination etc. The number of possible RNA modifications described to date exceeds 170 (1), with the presence on all RNA species and tRNAs being the most extensively modified (2). Modified nucleotides can affect the physiology of RNA in various ways, including secondary structure stabilization, and signaling through specialized proteins binding to modified RNA. In general, RNA modifications are involved in every aspect of mRNA metabolism, including regulation of mRNA splicing (3), stability of the transcripts (4, 5), translation efficiency (6, 7), and localization between different intracellular compartments (8, 9). The functions of non-coding RNAs are also strongly regulated through RNA modifications (10). For example, pseudouridylation of spliceosomal snRNAs (small nuclear RNA) is essential for spliceosome formation and efficient splicing (11, 12), and N6-methyladenosine (m6A) on lncRNA (long non-coding RNA) AGAP2-AS1 promotes its degradation, thus preventing the sponging of miR-424-5p which, in turn, regulates the expression of AKT3 and cell proliferation in keratinocytes (13).

Enzymes and proteins that interact with RNA modifications can be roughly divided into three categories: (a) writers catalyze the inclusion of modifications, (b) readers recognize and bind them, and (c) erasers remove them. While the functions of the writers and erasers are obvious, the readers have various modes of action, such as direct implementation of certain catalytic activity like splicing (14) on the modified RNA, recruitment of other proteins that, in turn, exert their function (15), protection and stabilization of modified transcript (16). Considering the potential of RNA modifications to affect the cellular physiology, it is expected that the alterations in RNA modifications and expression of RNA modification-related proteins arise as disease markers or targets for the development of novel therapeutic agents (17, 18).

Allergy is an overreaction of the immune system to harmless substances, such as pollen, pet dander, or certain foods. Common allergic diseases include hay fever, atopic and contact dermatitis, asthma and food allergies (19–21). The development of allergic diseases is a complex process, where both, innate and adaptive immune responses are involved. As the mechanisms of allergic diseases are discussed well in numerous other review articles (19–23), we hereby provide only a brief basic background. The development of many allergic conditions starts with sensitization, when antigen presenting cells (APCs), such as dendritic cells (DC) or Langerhans cells (LC) encounter and present allergen-derived antigens to naïve T helper (T_H_) cells, which then initiate T cell and B cell-based immune memory to the allergen. These allergic conditions are characterized by skewed systemic immune responses towards type 2 immunity, in which T_H_2 cells, high levels of IgE antibodies, but also activated type 2 innate lymphoid cells (ILCs) and eosinophils play important roles. This kind of immune responses are often characteristic to atopic dermatitis, allergic asthma, allergic rhinitis and food allergies (22, 23). When these allergic conditions are ongoing, the repeated encountering of allergen derived antigens associated with IgE may interact with the high-affinity receptors for IgE (FcεRI) present in mast cells (24), eosinophils, basophils, leading to initiation of allergic reaction and tissue inflammation (19, 25, 26). In case of some other allergic diseases, such as allergic contact dermatitis, mainly different types of T cells are responsible for immune memory (21, 27). In addition, neutrophils, macrophages, regulatory T and B cells, epithelial barrier integrity, epithelial cell responses, viral infections and microbiota participate in the development of all allergic conditions (28–30). As a result, persistent changes, also occurring in innate immune and epithelial cells, can be taken together as innate immune memory (31). Thus, molecular mechanisms of allergic diseases are highly complex and heterogeneous even within the same condition, while different allergic diseases may simultaneously have overlapping immunopathologies (32). This results also in large differences in treatment responses with poor outcome in certain cases, both with classical and more general, as well as novel and more specific biological treatments (33).

Recent studies highlight that both the genetic and epigenetic factors play role in the development and severity of allergic diseases (34). Any dysregulation in gene expression may result in imbalanced immune response. While numerous studies have addressed the role of epigenetics, transcription factors and other regulatory mechanism, such as miRNAs in the development of allergic diseases, the involvement of RNA modifications in allergy remains largely unexplored (34, 35). Still, the established knowledge of their role in immune responses creates a strong basis for speculation about potential implications in the development of allergic conditions, which we will give an overview in next chapters.

## RNA modifications and their functions

**Figure 1:**
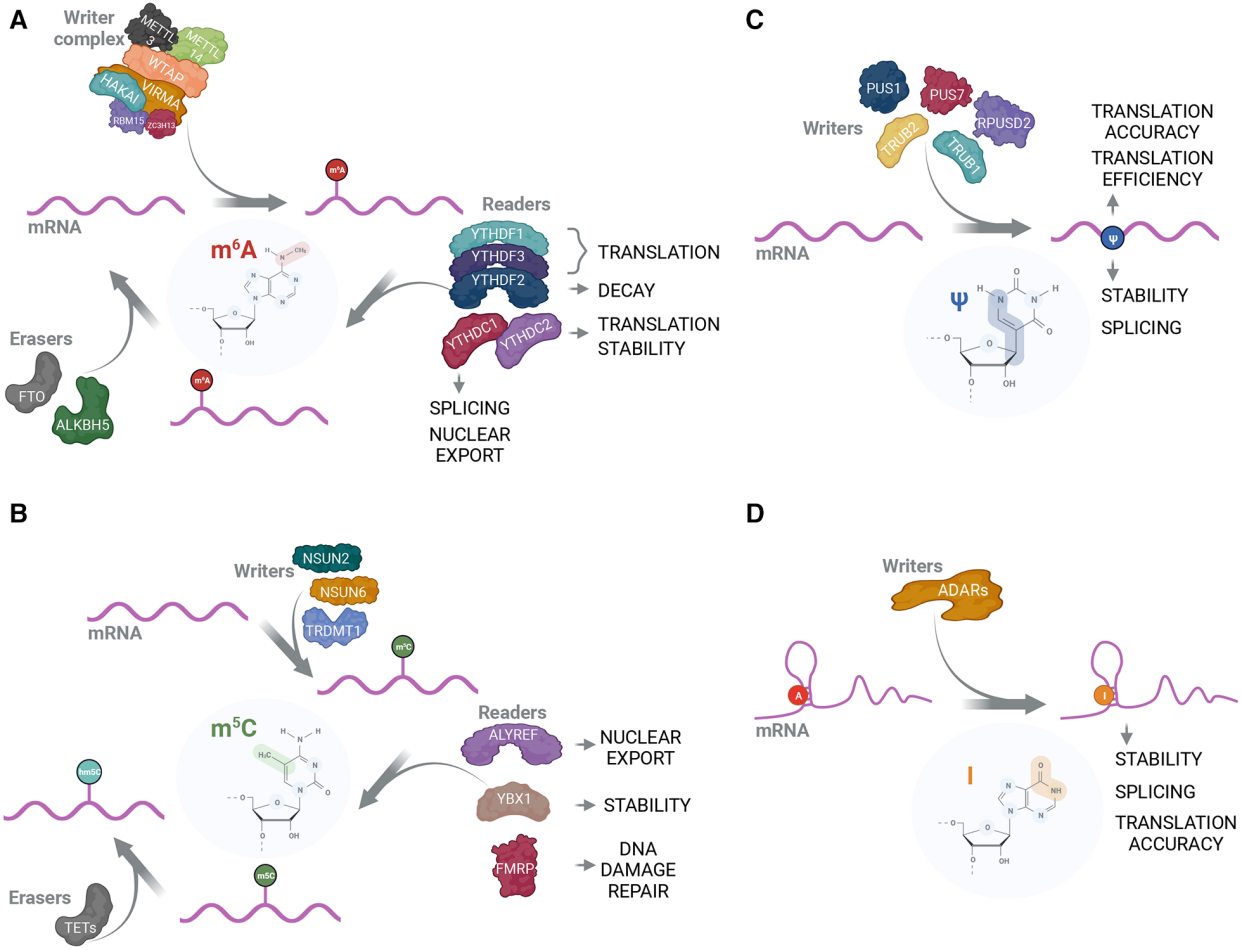
The protein interactome and functions of mRNA modifications. (**A**) m6A is positioned on mRNA by the methyltransferase complex consisting of catalytical subunit that includes METTL3 and METTL14, and structural subunit that includes WTAP, VIRMA, HAKAI, RBM15 and ZC3H13 (36–39). Subsequently, m6A-modified mRNA can be recognized by reader proteins that exert their functions in a variety of RNA-related processes (3, 16, 40–42). The most prominent m6A-readers belong to YTH-domain containing proteins (4, 43–46). FTO (47, 48) and ALKBH5 (49) can act as m6A erasers by removing it from mRNA; (**B**) m5C can be installed by individual writers; for mRNA these are NSUN2 (8, 50), NSUN6 (51) and TRDMT1 (52). ALYREF (8), YBX1 (50) and FMRP (53) are reported to act as m5C readers and affect nuclear export, RNA stability and DNA damage repair, respectively. m5C can be modified into hm5C by TET-family proteins (53, 54); (**C**) Pseudouridylation is catalyzed by PUS family enzymes with PUS1, PUS7, TRUB1, TRUB2 and RPUSD2 using mRNA as a substrate (55–57). Due to the absence of known erasers, pseudouridine is considered irreversible. While Ψ is shown to be important in mRNA translation accuracy, translation efficiency, stability and splicing (55–57), no Ψ-readers have been reported; (**D**) ADAR proteins are capable of performing A-to-I editing on double-stranded RNA (58, 59). Inosine is typically recognized as G during translation, thus causing codon and potential amino acid change in nascent peptide (60). Additionally, inosine is implicated in RNA stability (61) and splicing regulation (62).

The majority of non-rRNA/tRNA RNA modifications described to date are base methylations, of which we give overview of those, which biological functions are described better (Figure 1). The most studied and most abundant mRNA modification is m6A. Numerous studies have shown the importance of m6A for RNA-related processes in every aspect of cellular physiology [reviewed in (63, 64)]. m6A is established on mRNA by the “writer” complex, consisting of two functional subunits, catalytical subunit of methyltransferase-like 3 and 14 (METTL3 and METTL14) (36), where METTL3 fulfills the methyltransferase function and METTL14 has a supportive role of enhancing METTL3 binding to the target sequence (37), and regulatory subunit of Wilms' tumor 1-associated protein (WTAP), KIAA1429 (VIRMA), Zinc finger CCCH domain-containing protein 13 (ZC3H13), RNA binding motif protein 15/15 paralog (RBM15/15B), and E3 ubiquitin ligase CBLL1 (HAKAI) (36) (Figure 1A). In addition to well-characterized writer complex, various reader proteins have been reported to specifically recognize m6A. Most prominent m6A readers belong to conserved YTH domain-containing protein family that consists of 5 members in human—YTHDF1, YTHDF2, YTHDF3, YTHDC1 and YTHDC2 (43–45). YTHDF1 and YTHDF3 are considered to enhance the translation of modified transcripts while YTHDF2 promotes the degradation of m6A-enriched mRNAs (4). These readers fulfill their functions through recruiting translation initiation and mRNA decay machineries to target transcripts, respectively (15, 65, 66). YTHDC1 is involved in regulation of alternative splicing through recruiting of splicing factor SRSF3 to target sequences (67). Interestingly, YTHDC2 is the only YTH family protein that also has a helicase domain (68, 69) that is required for resolving mRNA secondary structures and, thereby, promotion of translation (70). Besides YTH-domain family proteins, a variety of other m6A-readers have been described, including IGF2BP (16) and certain HNRNP proteins (3). Two m6A-eraser proteins are also well-characterized. These are fat mass and obesity-associated protein (FTO) (47) and a-ketoglutarate-dependent dioxygenase alkB homolog 5 (ALKBH5) (49) (Figure 1A).

Another prominent methylation RNA modification is 5-methylcytidine (m5C) that is deposited by NSUN (NOL1/NOP2/SUN RNA methyltransferase) family proteins (71) (Figure 1B). m5C is important for mRNA stability and subcellular transport—these functions are fulfilled through the recognition of m5C-modified mRNAs from m5C readers, Y-box binding protein 1 (YBX1) that recruits ELAVL1 mRNA stabilization factor (50), and export factor ALYREF (8), respectively. Additionally, m5C is implicated in processing of small non-coding RNAs (72). Unlike m6A, m5C cannot be completely erased but converted into 5-hydroxymethylcytidine (hm5C) through the action of ten-eleven translocation (TET) family proteins (54).

Out of non-methylation RNA modifications, pseudouridines (Ψ) are strongly enriched in stable non-coding RNA species such as rRNA and tRNA where their main role is stabilization of secondary structure. Ψs are also present in other types of RNA, although with considerably decreased abundancy. Nevertheless, Ψ levels on mRNA can change significantly as well as expression and localization of pseudouridine synthases (PUS) (55, 56, 73) (Figure 1C). Cellular mRNA pseudouridylation may play a role of adaptation mechanism as in response to starvation (56) and oxidative stress (73). Ψ is also implemented in the regulation of splicing and alternative splicing (55) and can reduce the immunogenicity of the transfected RNA (24, 74). Another well-characterized function of Ψ is the promotion of stop-codon readthrough (75). Additionally, dependent on the context, Ψs either positively or negatively affect the translation efficiency of the given mRNA (24, 76, 77). Mentioned Ψ features are widely used in mRNA-based vaccines where most uridines are replaced with either Ψs or methyl-1-pseudouridines (m1Ψ) (24, 76).

Inosine (I), a deamination product of adenosine, is another abundant RNA modification. It is catalyzed by adenosine deaminases acting on RNA (ADARs) (78–81) (Figure 1D). Inosine occurrence is a source of mismatching, as I base pairs with cytidine and is reverse transcribed as guanosine (82), and, therefore, has a strong influence on cellular regulatory processes through disruption or introduction of target sequences for certain enzymes or non-coding RNAs to bind (61, 62, 83). In a similar fashion, I can be a cause for amino acid substitution with protein function alteration as a consequence (84–86).

Considering the significance of RNA modifications for various regulatory cellular processes, recent research has extensively explored their implications in health disorders. Thus, RNA modifications and related proteins are strongly involved in cancer physiology as being markers for the disease, onco-suppressors or oncogenes, dependent on the type of cancer [reviewed in (18, 38)], neurological diseases [reviewed in (87)], immune system diseases [reviewed in (88)], cardiovascular diseases [reviewed in (17, 89)] etc.

## RNA modifications in immune cells and their potential involvement in allergic diseases

Although the molecular mechanisms underlying the allergic diseases are extensively studied, the role of RNA modifications remains unexplored and no direct link between RNA modifications and allergic diseases has been established. Therefore, here we only focus on those cell types, about which there is available research results correlating their function with RNA modifications. These include DCs, macrophages, T and B cells, and mast cells. While the implication of several other cell types, such as basophils, neutrophils, eosinophils and ILCs in allergy is widely recognized (25, 90), these cell types have not been studied in the context of RNA modifications, and not discussed in this review.

### DCs and macrophages

DCs and macrophages are involved in the development of allergic inflammation as APCs, and in the regulation of inflammatory responses (91). In case of some lineages, DCs and macrophages have common precursor, as well as there is available a well characterized *in vitro* model to differentiate them from peripheral blood CD14+ monocytes. Therefore, we discuss the effects of RNA modifications in these cell types here together (92, 93). In macrophages, m6A has been shown to participate in the activation of macrophages. Thus, in the absence of m6A macrophages fail to activate in response to lipopolysaccharide (LPS) stimulation. METTL3-deficiency resulted in higher expression of IRAKM (IL-1 receptor-associated kinase 3) and consecutive supression of TLR (Toll-like receptor) signaling pathway (94). Another study, however, reports on overactivation of macrophages in response to LPS stimulation due to m6A-deficiency showing that METTL14 depletion leads to inability of YTHDF1 to bind SOCS1 mRNA followed by diminished activity of SOCS1 and subsequent upregulation of TLR4/NF-kB pathway (95). In support of previous study, depletion of YTHDF2 was similarly reported to cause overactivation of macrophages with upregulated NF-kB and MAPK pathways resulting in overexpression of pro-inflammatory cytokines (96). Depending on the stimuli, activated macrophages can be roughly divided between two states, M1 and M2—pro-inflammatory and anti-inflammatory, respectively (93). METTL3 KD causes the shift in macrophage polarization towards M2 as in the absence of m6A STAT1 mRNA has lower stability and expression that negatively affects M1 polarization (97). Additionally, METTL3-loss leads to decrease in the translation efficiency of SPRED2, the enhancer of NF-kB and STAT3 activation, resulting in elevation of M2 macrophages (98). m6A was also shown to be crucial for macrophage-induced inflammation (95, 99) and pyroptosis (100). Participation of ADAR1 in the regulation of macrophage polarization has been confirmed as well. ADAR1 overexpression promotes M2 polarization in miR-21-dependent manner. A-to-I modification in miR-21 precursor leads to uregulation of Foxo1 (forkhead box protein O1) and overexpression of IL-10 (101). Interestingly, in DCs, depletion of inosine RNA modification writer, ADAR1, caused severe dysregulation in DC differentiation. Thus, certain subpopulations of DCs, such as CD8^+^/CD103^+^ DCs that are able to activate CD8^+^ T cells in the context of major histocompatibility complex (MHC) class I (102), are majorly lost in response to I deficiency (103). This could be especially relevant for later stages of non-IgE-mediated allergic disorders where CD8^+^, activated in the context of MHC class I, are the main contributors to the disease phenotype (27). RNA modification m6A is also strongly involved in DC physiology. Normally, m6A levels increase during the maturation of DCs and signal for YTHDF1 to promote the translation of modified mRNAs. METTL3 depletion impairs the maturation of DCs that reflects in downregulated expression of TLR signaling adaptor TIRAP, co-stimulatory molecules CD40, CD80 and IL-12 (104). Additionally, METTL3 knock-out (KO) may lead to decreased production of MHC class II and interferon γ (IFNγ) (105). Taken together, m6A deficiency strongly reduces the ability of DCs to activate T cells (104, 105).

### T cells

Similar to its role in DCs, m6A is implicated in the regulation of T cell differentiation (106), including the modulation of T_H_1/T_H_2 ratio (105, 107). m6A eraser ALKBH5 is upregulated in activated CD4^+^ T cells to increase the stability of synthesized IFNγ and C-X-C motif chemokine ligand 2 (CXCL2) mRNAs (108). Depletion of METTL14 leads to dysfunctional differentiation of naïve T cells into regulatory T cells (Treg) as well as attenuated capacity of Tregs to restrain inflammation (109). One of the possible reasons could be the decreased expression of retinoic acid–related orphan receptor (RORyt) (109) that is crucial for the functioning of certain subset of Tregs (110). Another study reports on decrease of Treg suppressive function through the deactivation of crucial IL-2-signal transducer and activator of transcription 5 (STAT5) signaling by overexpression of suppressor of cytokine signaling (SOCS) family proteins, caused by METTL3 depletion (111). As for the other RNA modifications, ADAR1 is crucial for T cell maturation (112) and NSUN2-catalyzed m5C on IL-17A mRNA promotes the translation of IL-17A in T cells (113).

### B cells

During IgE-mediated allergic reaction, activation of type 2 immune response conducted by T_H_2 cells, promotes immunoglobulin class-switch recombination in B cells to activate the production of IgE antibodies. Maturation of B lymphocytes is a complex multi-stage process and depletion of ADAR1, performed on different stages, dramatically impairs consecutive development and strongly decreases the capacity of B cells to get activated by T cells (114–116). Deficiency in m6A, caused by the depletion of METTL14, also strongly impacts the transition between stages during the maturation of B cells. Blockage of transition events can be explained by defective regulation of expression of critical genes since the transcripts that lack m6A are not recognized by YTHDF2 and not targeted for degradation (117). Class-switch recombination, induced by T cells, is mediated through m6A. RNA exosome, recruited by YTHDC1 for correct processing of nascent m6A-modified immunoglobulin switch region transcripts brings in the factors, essential for further progress of DNA recombination (118).

### Mast cells

Mast cells and, to some extent, basophils are the major regulators of IgE-mediated allergic reaction. Aggregation of allergen-derived antigen-specific FcεRI receptors on the surface of MCs and basophils induces degranulation and consecutive inflammation, followed by the manifestation of symptoms, characteristic for allergic reaction (119). Comprehensive analysis of m6A-related proteins in patients with respiratory allergic diseases showed negative correlation between the expression of METTL14 and m6A reader RBM15B, and MC infiltration rate (120). Moreover, m6A was shown to be important for the regulation of cytokine expression in mast cells. Thus, in the absence of METTL3, the stability of certain pro-inflammatory transcripts, especially IL-6, TNFα and IL-13 that are crucial for MC effector function representation (119), was significantly increased. At the same time, increased degranulation, as well as attenuated MC proliferation, was also observed (121).

## Conclusions and perspectives

**Figure 2:**
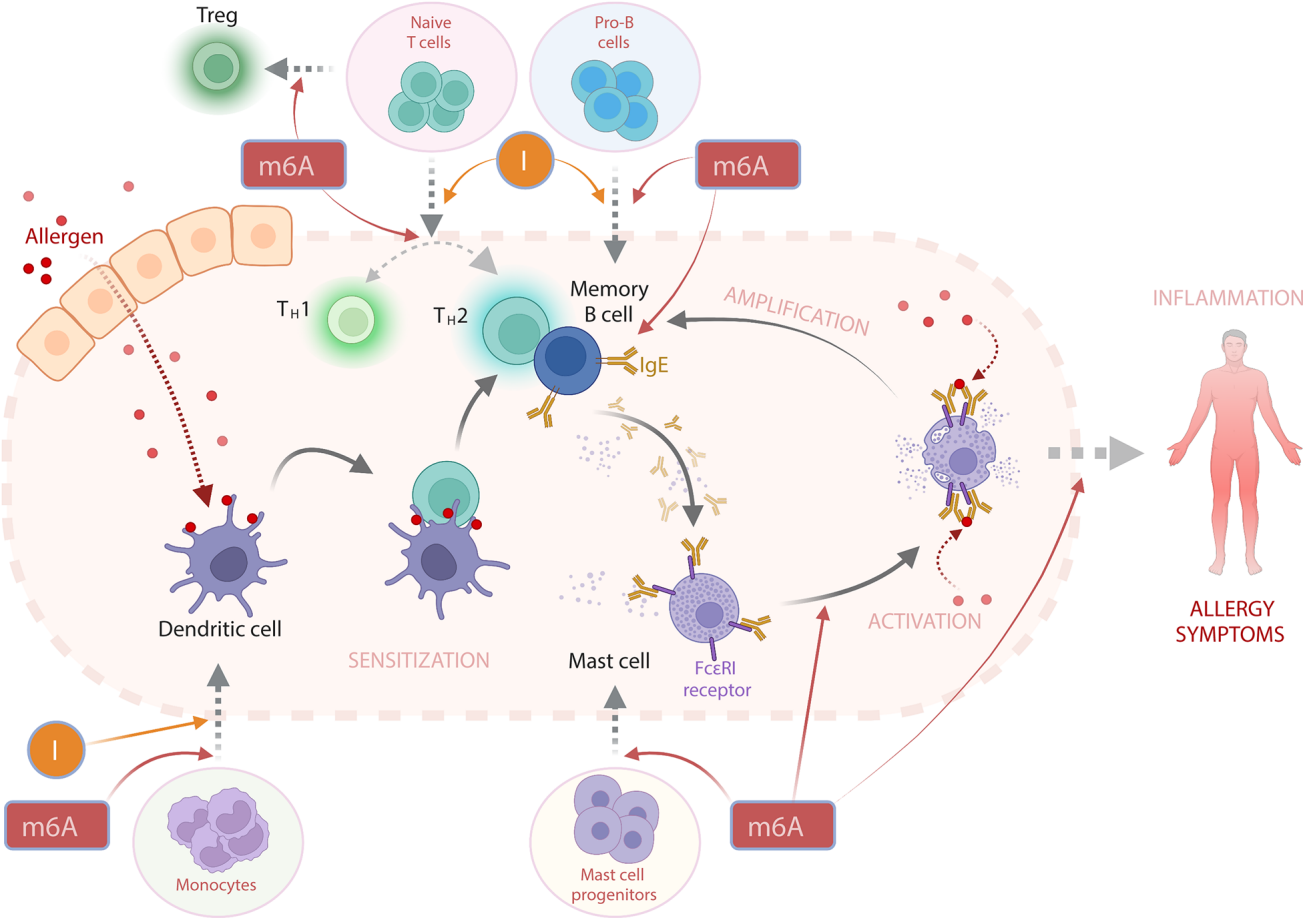
The potential involvement of RNA modifications m6A and I (inosine) in the activation of IgE-mediated allergic reaction. In a simplified view, an allergic response begins with the initial encounter with an allergen—a process known as sensitization. Here, antigens derived from the allergen are displayed on the surface of dendritic cells (DCs). This prompts the activation of T-helper 2 (T_H_2) cells, which subsequently stimulate B cells to produce allergen-specific Immunoglobulin E (IgE) antibodies. These IgE antibodies can attach to the IgE-specific receptor FcεRI on mast cells (MCs) and other cell types. Upon subsequent exposures to the same allergen, there is an overproduction of IgE in a phase termed “activation”, that results in the degranulation of MCs and the release of effector molecules, including histamine and various cytokines. These released cytokines can further enhance B cell activation, thereby amplifying the allergic response. The cumulative effect of these interactions triggers an inflammatory response and the emergence of allergy symptoms. While there are no direct reports of RNA modifications playing a role in the progression of an allergic reaction, it is plausible that m6A and inosine (**I**) could indirectly influence the allergic response's development. I is critical for the differentiation of DCs (103), T cells (112), and B cells (115). m6A is not only vital for the differentiation of above-mentioned cells (104, 106, 117) but is also implicated in regulating the T_H_1/T_H_2 cell balance (107), T regulatory (Treg) cell differentiation (109), MC differentiation and functioning of the latter (121).

RNA modifications play a pivotal role in cellular function and regulation, impacting numerous biological processes essential for health and disease. Allergic diseases result from aberrant immune responses, involving various cell types and a complex interplay of cytokines, chemokines, and other immune mediators, which all lead to changes in adaptive and innate immune memory. We propose that RNA modifications play a critical role in these processes through their known contributions to the development and function of macrophages, DCs, T cells, B cells, and mast cells as well as by other yet undescribed mechanisms. As an example, we have generated a model, how m6A influences the development of allergic inflammation (Figure 2). Still, while our understanding of the role of RNA modifications in the immune system is growing, it remains significantly limited, primarily due to the fact that the majority of current research focuses on m6A. This is a significant gap, considering that more than 170 distinct RNA modifications have been characterized to date (1). Other modifications, such as Ψ, m5C and inosine, among others, have been identified in various types of RNA, yet their functions, particularly in the context of immune system biology and allergic disorders, are largely unexplored. These less-studied modifications could be just as important as m6A in immune regulation and could be involved in the pathogenesis of allergic diseases. Therefore, a more detailed understanding of the roles of RNA modifications, which biological functions are already somewhat known in immune system, as well as those of that are less known, would be important in insights into the pathogenesis of allergic disorders.

Furthermore, changes in RNA modification patterns could reflect disease status, progression, or response to treatment. Moreover, enzymes responsible for RNA modifications could be exploited as markers for disease (122–124) and potential therapeutic targets (18). For instance, small molecules modulating the activity of methyltransferases (125) or demethylases (126) could be used to manipulate RNA modification levels, thereby influencing immune responses and potentially alleviating allergic symptoms. Current treatments for chronic allergic disorders primarily aim at managing symptoms and reducing exposure to allergens. Common therapeutic strategies include antihistamines, corticosteroids, and in some cases, allergen-specific immunotherapy. However, these treatments often have limitations, including side effects and variable patient response (127). Similarly, novel biological treatments are not equally efficient in all of the patients (33). Targeting RNA modifications may potentially offer a novel approach for allergy treatment. By precisely modulating immune responses at the RNA level, this strategy could provide more specific and efficient treatment, with potentially fewer side effects. Combinatorial therapy integrating traditional anti-allergy drugs, biological treatments and novel therapeutics targeting RNA modifications might further optimize treatment outcomes and improve patients' quality of life. Another strategy presumes usage of RNA modifications and related proteins as powerful tools for biomedical research and therapeutic development. A key example of this is the use of pseudouridines in mRNA vaccines, such as those developed for COVID-19 (76). Pseudouridine-modified mRNAs show enhanced stability and translation efficiency, leading to stronger protein expression. Additionally, the inclusion of pseudouridine helps to evade recognition by the immune system, reducing the risk of an unwanted immune response against the therapeutic mRNA (24, 74). This exploitation of a natural RNA modification has revolutionized vaccinology, enabling the rapid development of effective vaccines in response to emerging infectious diseases (76).

In conclusion, we propose that RNA modifications may play crucial roles in allergic disorders, impacting disease pathogenesis, diagnosis, and treatment. Continued exploration in this field can uncover new diagnostic markers and therapeutic targets, potentially revolutionizing allergy management and offering hope for millions of individuals suffering from these conditions.
